# Mastering of Filled Rubber Strength beyond WLF: Competition of Temperature, Time, Crack Deflection and Bond Breaking

**DOI:** 10.3390/polym14040765

**Published:** 2022-02-16

**Authors:** Jan Plagge

**Affiliations:** School of Mathematics and Natural Sciences, Bergische Universität Wuppertal, Gaußstr. 20, D-42097 Wuppertal, Germany; plagge@uni-wuppertal.de

**Keywords:** filled rubber, structure–property relationships, reinforcement, nano-fillers

## Abstract

Tensile strength is an important indicator for elastomer toughness. However, in filled materials, its dependency on temperature and time appears to be poorly understood. We present experimental tensile data of carbon-black-filled ethylene propylene diene rubber at different temperatures. Tensile strength vs. filler loading exhibited a temperature-dependent S-shape and could be rescaled to collapse onto a single master curve. A model based on the extension of the time–temperature superposition principle, crack deflection, and breakage of covalent bonds is proposed. It successfully predicted the behavior of tensile strength due to the change of the filler particle size and filler amount, temperature variation, and deformation speed typically found in the literature. Moreover, stress relaxation during temperature ramp-up was reproduced correctly. Altogether, the successful modeling suggests that the true toughness of rubber (e.g., chemical bonds) becomes important once enough crack-screening filler is present.

## 1. Introduction

Cross-linked, pure rubber is mechanically too weak for most practical applications. The addition of nanoscopic filler particles improves the tensile strength and abrasion resistance by up to an order of magnitude. For this reason (among others), almost all technical goods are highly filled. An exemption may be strain-crystallizing polymers, most importantly natural rubber (NR) [1,2,3]. Nanoscopic carbon black was the first and is still the most-used reinforcing filler for rubbers. Its effect on the properties of the rubber has been the subject of research for more than 100 years and is covered in many review articles [4,5,6,7,8,9,10]. While there have been many attempts to model the actual stress–strain relationship of filled rubber on a physical basis [11,12,13], there are comparatively few works dealing with the ultimate properties of the material. Most approaches rely on the idea that tensile strength is limited by the largest “crack nucleus” present in the material, e.g., non-dispersed filler agglomerates [14]. This effect can also be studied by including glass beads of a defined size [15]. An older, but comprehensive overview about different fracture mechanisms was provided by Gul [16]. Recently, Watanabe et al. observed a crack in situ using transmission electron microscopy [17]. The crack progressed in a stick–slip manner by delaminating rubber from the (non-treated) silica surface.

Unfilled rubber is known to obey the time–temperature superposition principle, e.g., formulated via the Williams–Landel–Ferry (WLF) equation. This holds true for the ultimate properties, especially tensile strength as well [18,19]. However, for filled rubbers, the WLF theory is generally not always fulfilled [4]. To the best of the authors knowledge, there is no predictive physical theory to explain the increase in tensile strength due to fillers and relating it to temperature and time. It is worth noting that tensile strength is just one of many possible indicators for “reinforcement”. For example, surface deactivation (graphitization) of carbon black reduces the tensile strength by about 10–20%, but the overall shape of the stress strain curve changes dramatically, as does the abrasion resistance (three-fold decrease) [20,21].

The present paper aims to provide a simple theory that generalizes the tensile strength time–temperature superposition principle to filled rubbers. It is based on (i) the assumption that defect-induced cracks or load peaks are screened at filler particles and (ii) that highly filled materials fail mainly due to the breakage of (sulfur) bonds in the bulk polymer phase. The paper is structured as follows. First, we discuss the experimental data on the tensile strength of ethylene propylene diene monomer (EPDM) rubber filled with varying amounts of carbon black at different temperatures. The results stimulated the following theory, which was then successfully applied to the data. Finally, some theory predictions are shown.

## 2. Experimental Section

The samples consisted of an amorphous-type EPDM (Keltan™ 4450), 10–60 phr N339-grade carbon black, 3 phr zinc oxide, and 1 phr stearic acids. No aging protection (e.g., antioxidants) or other chemicals were included in the compound to keep it as simple as possible. The ingredients were mixed in a Rheomix 3000E internal mixer with Banbury rotors at the laboratory scale. Carbon black was added in two steps. Mixing was finished after the torque was observed to be constant for 3 min, after around 15 min of total mixing time. The vulcanization system consisted of 1.05 phr sulfur and 1.40 phr CBS, which was added subsequently using a roller mill. The samples were vulcanized into 2 mm-thick plates in a heated press at 160 °C and 280 bar. The curing time was defined to be the time required to reach 90% of the maximum torque in a vulcameter ($t_{90\%}$ time), plus 1 min per mm sample thickness to account for thermal diffusion. The samples were exactly the same as the labeled ref${\;}^{*}$ in [20], but with varying amounts of carbon black. Stretching was performed in a Zwick Z010 stretching machine on a standardized ISO 527-2 Typ 5A (S2) (flat dumbbell/dog bone) test specimen in a heating chamber at a strain rate of (4.5±0.7)%/s. At least 5 samples per temperature and filler loading were tested. If fewer data points are given, the corresponding samples failed during clamping in the stretching machine.

## 3. Discussion of the Experiments

**Figure 1:**
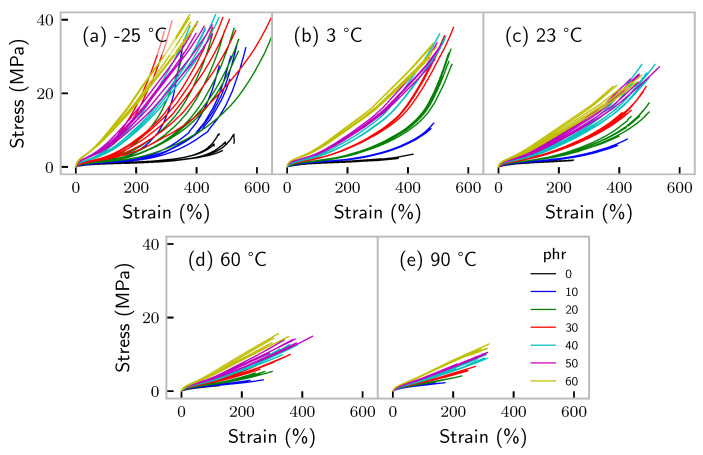
Results of tensile tests performed on carbon-black-filled EPDM test specimen at a constant deformation rate and varying temperatures. Strain measurement at −25 °C is not reliable.

Individual curves of the tensile tests are presented in Figure 1. Note that at −25 °C, the glue of the strain-tracking reflecting points, used for optically measuring strain was too hard to follow the samples’ deformation, explaining the seemingly large deviations of the results in terms of strain. This did not affect our results, as we were dealing exclusively with (maximum) stress. At 90 °C, it was not possible to measure the unfilled sample, because it was already destroyed during clamping. This holds as well for the majority of the unfilled samples at 60 °C.

**Figure 2:**
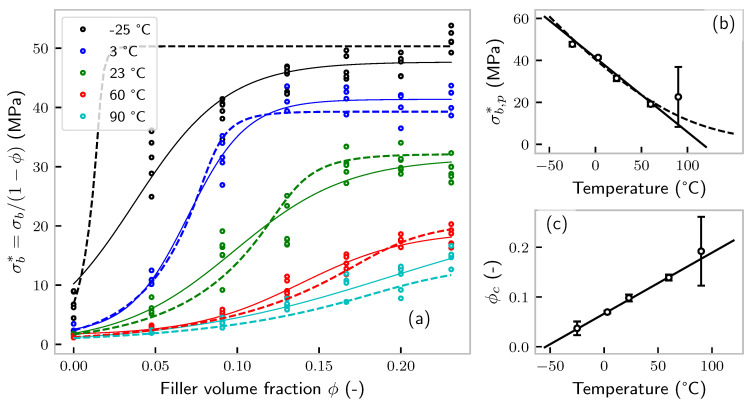
Evaluation of the tensile data shown in Figure 1. (**a**) Tensile strength of the polymer phase vs. filler volume fraction at different temperatures. Dots represent experimental data, solid lines the fit of Equation (2), and dashed lines the model fit according to Equation (13). Solid lines: Tanh fit. Dashed lines: model fit. (**b**) Plateau value of $\sigma^{*}$ vs. temperature. The dashed line is that predicted by Equation (11). (**c**) Critical volume fraction (obtained as the turning point of the solid lines in (**a**)) vs. temperature.

When filler is present the relative amount of the polymer is reduced. For this reason, we subsequently work with the effective tensile strength of the polymer matrix $\sigma_{b}^{*}=\sigma_{b}/\left(1-\phi \right)$. In the latter formula, $\sigma_{b}$ is the tensile strength (“engineering stress”) and ϕ represents a lower bound of the filler volume fraction, as a highly structured filler could shield additional rubber from participating in the deformation (occluded rubber) [22]. The filler volume fraction is calculated via:

(1) $$\begin{array}{c} \phi =\frac{\left(\mathrm{phr}\;\mathrm{filler}\right)/\rho_{\mathrm{filler}}}{100/\rho_{\mathrm{polymer}}+\left(\mathrm{phr}\;\mathrm{filler}\right)/\rho_{\mathrm{filler}}} \end{array}$$

where phr means “(mass) parts per hundred rubber” and $\rho_{\mathrm{filler}}\approx 2\;g/{\mathrm{cm}}^{3}$ and $\rho_{\mathrm{polymer}}\approx 1\;g/{\mathrm{cm}}^{3}$ are the approximate mass densities of carbon black and polymer, respectively. The tensile strength (maximum stress), corrected by the volume occupied by filler $\sigma_{b}^{*}$, vs. the filler volume fraction ϕ is shown in Figure 2a.

It appears that, at least in the filler-volume-corrected representation, the curve exhibits an S-shape with a plateau of tensile strength for high amounts of filler. Surprisingly, this was violated at −25 °C, but this is close to the glass transition of the EPDM at −50 °C, where the split into a force-carrying soft polymer matrix and rigid filler particles is probably not meaningful anymore. Even though one might be tempted to relate the steep increase of the tensile strength to the filler percolation threshold, it shall be highlighted that the latter does not depend significantly on temperature [23], while the former obviously does. Thus, the often observed increase in tensile strength and percolation threshold at room temperature has to be regarded as a random coincidence. At this point, a few words on the generality of this finding are warranted. Very similar results were found as early as 1951 by Parkinson on carbon-black-filled SBR rubber [24]. Reference [25] published extensive data on the dependence of the tensile strength on the temperature and the carbon black type and loading. For SBR, the results were again similar to our findings, even though it has to be taken into account that the authors used “true” stress with respect to the actual (not original) cross-section. Unsurprisingly, they obtained a different picture for NR-based compounds, where strain crystallization guarantees a high tensile strength even at low filler loadings. The authors found a very similar trend, if the filler loading was replaced by the filler surface area. Neogi et al. found similar effects for SBR as well [26]. They also provided temperature-dependent data on NR-based compounds, whose characteristics became similar to those of SBR at high temperatures (when strain crystallization is suppressed). In a very useful and extensive work, Wang et al. investigated the tensile strength (among other aspects) of carbon-black-filled SBR and nano-zinc-oxide-filled EPDM [27]. They found the usual S-shaped dependency of tensile strength vs. carbon black loading. A similar trend holds for zinc oxide, despite its supposedly bad interaction with the polymer. Moreover, a super-linear relation between the calculated particle distance and tensile strength was found. They attributed the upturn in the S-shape to a percolation threshold (see the comment above). Choi et al. investigated the effect of carbon black and silica (not surface modified) of a similar specific surface area (119 vs. $175\;m^{2}/g$) on the properties of SBR [28]. Tensile strength vs. filler loading evolved surprisingly similarly and peaked at about 40 phr for both types. This is especially noteworthy, as all other mechanical properties (viscosity, elongation at break, modulus, abrasion resistance) were dramatically different. Moreover, a varying amount of coupling agent (Si69) to a SBR-silica compound has been shown to have a small effect on tensile strength [27]. Surface deactivation of carbon black has a rather small effect on tensile strength as well [20].

We conclude that the observed behavior seems to be universal to rubbers without special structural features, e.g., strain crystallization, filled with particles of a high specific surface area that do not necessarily have to exhibit a strong interaction with the polymer.

For an analysis of the curves, we fit the data with the purely empirical function:

(2) $$\begin{array}{c} \sigma_{b}^{*}=a+\frac{b}{2}\tanh\left(\frac{2c}{b}\left(\phi -\phi_{c}\right)\right)+\frac{b}{2} \end{array}$$

where the plateau value can be calculated as $\sigma_{b,p}^{*}=a+b$. The latter is plotted vs. temperature in Figure 2b. Except the 90 °C measurement, which has a high uncertainty in the plateau value, the data is perfectly linear. By extrapolation, we find that the plateau stress became zero at 114 °C. However, even though sulfur-cured rubbers are known to fail early at temperatures above around 100 °C, they will certainly be able to still bear a small load. Thus, a deviation from linearity can be expected (see below).

**Figure 3:**
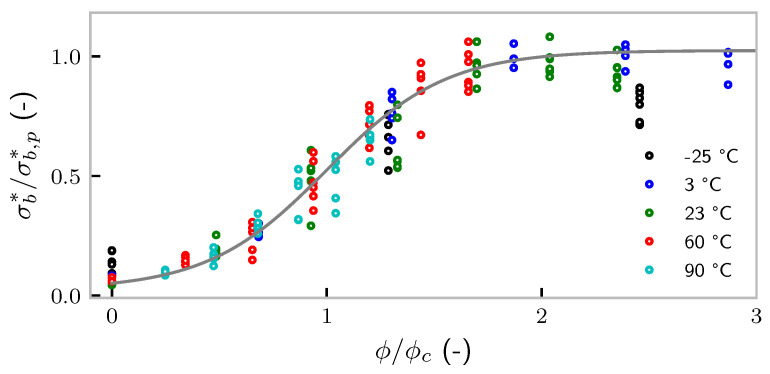
Data taken from Figure 2, but rescaled with respect to $\phi_{c}$ and $\sigma_{b,p}$. The grey line is a tanh-function as a guide for the eyes.

The turning point of the curve, denoted $\phi_{c}$, is shown in Figure 2c. Again, a perfectly linear behavior is found. $\phi_{c}$ vanishes at the (extrapolated) temperature −54 °C, which is rather close to the glass transition temperature of the polymer (Keltan™ 4450) obtained from dynamic mechanical analysis as −47 °C [29] (at a frequency of 1Hz). This finding suggests that the carbon black content required for a certain tensile strength is strongly influenced by the glass transition temperature. Given these surprising results, we rescale the data by $\phi_{c}$ and $\sigma_{b,p}$. The result is shown in Figure 3. Except the −25 °C measurement, all the data collapse onto a single line. Apparently, there is a seemingly simple relation among ϕ, *T* (and eventually, the deformation rate), and tensile strength.

## 4. Theory

Gent et al. showed that the tearing and breaking energy of unfilled SBR can be mastered with respect to time and temperature using the well-known Williams–Landel–Ferry (WLF) equation [30]. Similarly, Smith showed, on SBR as well, that this procedure can also be applied to tensile strength for samples at different temperatures, which are stretched at varying speeds [19]. Greensmith performed extensive tests on filled and unfilled SBR rubber with deformation rates between roughly 0.1%/s and 2000%/s and concluded that the results can be generally traced back to the viscoelastic nature of the material, even though he admitted that the matter appeared more complex for filled rubbers [31].

Thus, our starting point for unfilled rubbers shall be to express the tensile strength $\sigma_{b}$ as a function *f* of a reduced time $t/a_{T}$:

(3) $$\begin{array}{c} \sigma_{b}=f\left(t/a_{T}\right)≃{\left(t/a_{T}\right)}^{-\alpha } \end{array}$$

where the last term assumes a power-law representation in which $\sigma_{b}$ decreases with increasing temperature (α>0). Moreover, we introduced the famous WLF parameter:

(4) $$\begin{array}{c} \log_{10}\left(a_{T}\right)=-\frac{c_{1}\cdot \left(T-T_{0}\right)}{c_{2}+\left(T-T_{0}\right)} \end{array}$$

For our compound, these parameters were evaluated in previous publications as $c_{1}=1.13$, $c_{2}=85.3\;K$, and $T_{0}=23{\;}^{{}^{\circ}}C=276.15\;K$ and proved to be independent of filler surface treatment, viz. it can be expected that they represent the pure polymer matrix [20]. Our experiments were performed at a constant stretching rate; thus, in Equation (3), we set t=const. and arrived at $\sigma_{b}∼{\left(1/a_{T}\right)}^{-\alpha }$. The naturally arising question now is about the proportionality constant to the rate factor $1/a_{T}$, which already includes the full temperature dependency for an unfilled material. In the absence of specific effects, most prominently strain crystallization in natural rubber, unfilled rubbers have a far lower tensile strength than their filled counterparts. Thus, it appears reasonable to assume that there are always defects of unspecified origin in the material (e.g., cross-link clusters/heterogeneities, residual processing aids, zinc/stearate particles, etc.), which may induce stress peaks, which grow into microscopic, potentially catastrophic, cracks and are sampled at the typical timescale (given $∼a_{T}$) of the material. We call this the “defect rate”, which is assumed to be a property of the rubbery phase only (this assumption may be questioned for filled materials, where filler generally helps to disperse ingredients, but introduces undispersed filler aggregates at the same time), and we arrive at:

(5) $$\begin{array}{c} \sigma_{b}∼{\left(r_{d,0}/a_{T}\right)}^{-\alpha }. \end{array}$$

where we introduce $r_{d,0}$ as the “defect rate” at the reference temperature. After initiation, the crack may grow up to the rupture of the material. Even though we speak of a “crack” in the remainder, the discussion also holds equivalently for stress peaks, which might evolve into nuclei of cracks.

In the case of filled rubbers, there is a possibility that the crack tip (stress peak) hits the filler’s surface. Its energy may either be absorbed (e.g., because the filler acts as a giant cross-link and allows transferring the energy to many neighboring chains) or (partially) deflected and may hit another filler particle. To some extent, the situation is similar to scattering. Thus, the probability of the crack surviving a path of length $l_{c}$ can be assumed to decrease, on average, $∼\exp(-\sigma \eta l_{c})$ (repeated scattering). σ and η represent the scattering cross-section and number of filler particles, respectively. With $\sigma =v^{2/3}$ and η=ϕ/v, we arrive at:

(6) $$\begin{array}{c} \sigma_{b}^{*}=\frac{\sigma_{b}}{1-\phi }∼{\left(\frac{r_{d,0}}{a_{T}}e^{-\phi \frac{l_{c}}{v^{1/3}}}\right)}^{-\alpha }. \end{array}$$

where *v* is the volume of a filler particle and ϕ is the filler volume fraction. We consciously omitted the units (Pa) of the scaling relation. This relation already coincides with many observations, i.e., a higher filler volume fraction induces a super-linear increase of tensile strength, as do smaller filler particles. The crack of length $l_{c}$ originates from random fluctuations, which grow with a certain velocity and eventually become a crack. An upper bound for the speed of growth is given by the speed of sound *c*. We thus assume $l_{c}∼c∼\sqrt{E}∼\sqrt{a_{T}}$ and set:

(7) $$\begin{array}{c} l_{c}=l_{c,0}\sqrt{a_{T}}, \end{array}$$

where we neglect the comparatively weak effect of entropy elasticity (E∼T) on the modulus *E*. Relation (7) is probably the weakest hypothesis of this work. Alternatively, one could imagine a decrease of critical crack length proportional to the tensile strength of the unfilled matrix $l_{c}∼a_{T}^{\alpha }$ with similar results.

(8) $$\begin{array}{c} \sigma_{b,d}^{*}∼{\left(\frac{r_{d,0}}{a_{T}}e^{-\phi \frac{l_{c,0}\sqrt{a_{T}}}{v^{1/3}}}\right)}^{-\alpha }=r_{d}^{-\alpha }. \end{array}$$

where we introduce the crack failure rate $r_{d}$.

**Figure 4:**
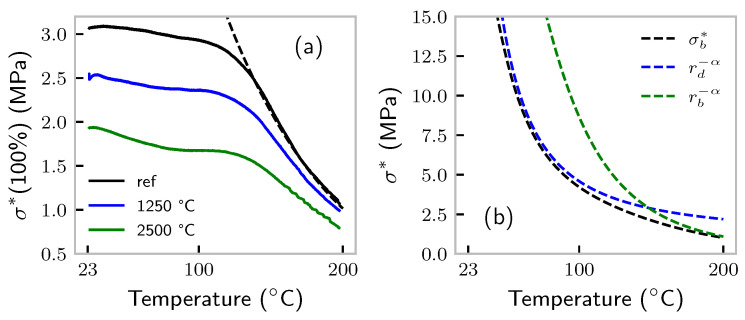
(**a**) Stress at 100% strain during a temperature ramp with 2 °C/min. “ref” corresponds to the 50 phr sample; see the text for the explanation. The dashed line represents a fit of Equation (13). (**b**) Split of Equation (13) into the contributions of defects and bond breaking.

Equation (8) suggests an infinite increase in tensile strength by the addition of filler. In contrast, Figure 2a clearly shows that the tensile strength saturates or even decreases above a certain amount of filler (and that it is not a simple consequence of correcting by the amount of filler (1−ϕ)). This plateau value is plotted vs. temperature in Figure 2b and, if the linear model is approximately correct, extrapolates to zero stress at around 120 °C. In the rubber industry, it is common knowledge that sulfur-cured vulcanizates should not be exposed to permanent temperatures above 100 °C. In fact, temperature scanning stress relaxation (TSSR) measurements of the used compound have shown a sharp decrease in modulus above 120 °C; see Figure 4 [32]. It thus appears reasonable to assume that the maximum achievable tensile strength is determined by the sulfur bonds between the network chains. The rate of bond breakage can be modeled by simple reaction rate theory as [33]:

(9) $$\begin{array}{c} r_{b}\left(\sigma_{b}\right)=r_{b,0}\;e^{-\frac{E_{b}-\sigma_{b}V}{RT}} \end{array}$$

with $r_{b,0}$ a rate constant to be specified, $E_{b}$ the potential barrier to surpass for sulfur bond breakage, and *V* a molar volume of the breaking entities. Equation (9) expresses that force on the chains tilts the potential landscapes and eventually leads to a “jump” over the potential barrier $E_{b}$. A similar expression was already used by Gul [16].

The tensile strength is then given by the self-consistent equation:

(10) $$\begin{array}{c} \sigma_{b,b}^{*}≃r_{b}^{-\alpha }={\left(r_{b,0}\;e^{-\frac{E_{b}-\sigma_{b}^{*}V}{RT}}\right)}^{-\alpha } \end{array}$$

whose solution can be expressed via the Lambert W function (product logarithm):

(11) $$\begin{array}{c} \sigma_{b,b}^{*}=\frac{RT}{\alpha V}W\left(\frac{\alpha V}{r_{b,0}^{\alpha }RT}e^{\frac{\alpha E_{b}}{RT}}\right). \end{array}$$

**Figure 5:**
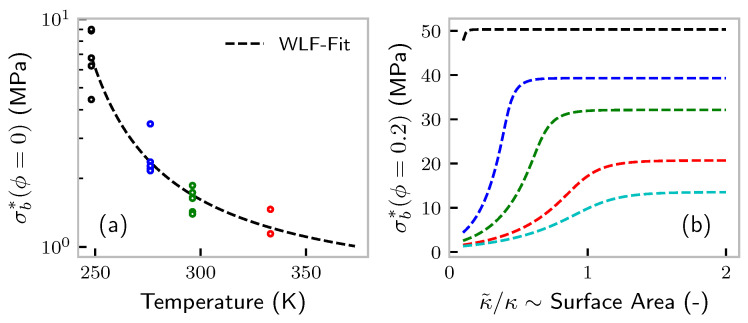
(**a**) Fit of tensile strength of unfilled samples to Equation (8). No data are available at 90 °C. (**b**) Effect of variation of κ around its fit value. The latter is directly proportional to the nitrogen surface area (NSA) of the filler. Color code as in Figure 2 (−25 (black) to 90 °C (cyan)).

One might argue that, in analogy to Equation (6), the WLF parameter $a_{T}$ should also scale the rate $r_{b,0}$. In the latter, $a_{T}$ was argued to originate from the idea that defects “pop up” with the frequency of polymer fluctuations. However, in Equation (9) the rate and its temperature dependence are not dependent on polymer fluctuations. By adding both rates, i.e., Equations (8) and (9), we obtain the self-consistent equation:

(12) $$\begin{array}{cc} \; & \sigma_{b}^{*}∼{\left(r_{d}+r_{b}\left(\sigma_{b}^{*}\right)\right)}^{-\alpha } \end{array}$$

(13) $$\begin{array}{cc} \; & ={\left(\frac{r_{d,0}}{a_{T}}e^{-\phi \frac{l_{c,0}\sqrt{a_{T}}}{v^{1/3}}}+r_{b,0}\;e^{-\frac{E_{b}-\sigma_{b}^{*}V}{RT}}\right)}^{-\alpha } \end{array}$$

which has to be solved by suitable numerical methods. However, most parameters can be determined on its asymptotic solutions, i.e., in the case of high crack rates $r_{d}\gg r_{b}$, it falls back to Equation (8). By including unfilled samples (ϕ=0), it is straightforward to determine the parameters α and $r_{d,0}$. The result of the fit of Equation (8) to the unfilled data is shown in Figure 5.

Equation (11) has three unknown parameters *V*, $r_{b,0}$, and $E_{b}$. Given that the data presented in Figure 2b are linear (not taking into account the data point with large uncertainty), a fit with three unknowns will be overparameterized. For this reason, one of the parameters has to be fixed. The most accessible appears to be the activation energy for bond opening. Reference [34] stated that the activation energy of rubber vulcanization, presumably the energy required to open an S8-ring, is around 146kJ/mol. This value was roughly confirmed by thermochemical calculations [35]. In another work, the activation energy was investigated on semi-efficiently cured, unfilled natural rubber samples using precise measurements of free sulfur content by Franck et al. and evaluated to be 99kJ/mol [36]. More recently, carbon-black-filled rubbers, including EPDM, were investigated by vulcametry. The activation energy was found to be around 72kJ/mol, only slightly dependent on the polymer and filler content [37]. Note that the latter range of activation energy was in full accordance with the rule of thumb that sulfur vulcanization time is halved if the temperature increases by 10 °C in the relevant range (130⋯170 °C). The scattering of numerical values showed that the activation energy probably depends on the amount and composition of vulcanization aids, e.g., accelerators. Moreover, we are interested in the debonding energy, and it is certainly a bold assumption born from the lack of knowledge to set activation and bonding energy equal. Nevertheless, we fixed $E_{b}=80\;\mathrm{kJ}/\mathrm{mol}$. The resulting curves do not differ too much if changed to $E_{b}=150\;\mathrm{kJ}/\mathrm{mol}$. The parameters *V* and $r_{b,0}$ can be robustly determined by fitting Equation (11) to the plateau value $\sigma_{b,p}$ shown in Figure 2b. The resulting fit is shown in Figure 2b as a dashed line.

**Figure 6:**
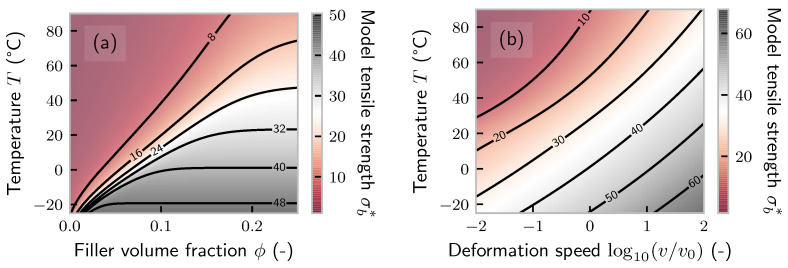
(**a**) Prediction of the model defined by Equation (13) and the parameters from Table 1 in ϕ−T space. (**b**) Prediction of the same model in v−T space.

Thus, only the parameter:

(14) $$\begin{array}{c} \kappa =l_{c,0}/v^{1/3} \end{array}$$

remains to be determined by fitting Equation (13) to Figure 2. The parameters can be found in Table 1, and the corresponding model fit is shown in Figure 2a as dashed lines. Figure 6a presents the solution of the model in the T−ϕ space.

As most fit parameters absorbed some kind of proportionality constant, it is hard to compare them to the literature data. The α parameter for unfilled SBR was found to be around 0.2 [30,38], probably deviating from our value because of the different polymer. A rough validity check can be carried out for *V*, i.e., the volume of the breaking entities. At a loading of 50 phr, the material used had a cross-link density determined by swelling and the Flory–Rehner theory of about $\nu =360\;\mathrm{mol}/m^{3}$ [20]. Even though the Flory–Rehner theory is not naively applicable to filled rubbers, the result is certainly precise enough for the sake of a rough comparison. Thus, the volume occupied by one mole of elastically active network chains is $1/\nu \approx 28\times 10^{-4}\;\mathrm{mol}/m^{3}$, being roughly in the same order of magnitude as *V* (especially when looking at the linear dimension instead of *V*). Another way to look at it is to express $\sigma \cdot V=f/b^{2}\cdot N_{A}b^{2}d=N_{A}fd$ as the product of force and distance *per chainf* and *d*. We additionally introduced the molecular cross-section $b^{2}$ of a chain and Avogadro’s constant $N_{A}$. Calculating $d=V/(N_{A}b^{2})\approx 7\;$Ågives an atomic distance (bond breaking), if b≈1nm is used.

We can check the qualitative validity of our model regarding the variation of the filler particle size. The work of Sambrook et al., especially Figure 2, tells us that an increasing nitrogen surface area (NSA) of the carbon black induces roughly the same effect as an increase in filler volume fraction [25]. In our model, the linear dimension of the filler particles $v^{1/3}$ enters via Equation (8). The nitrogen surface area is calculated as $\mathrm{NSA}∼\left(\mathrm{number}\;\mathrm{of}\;\mathrm{particles}\right)\cdot \left(\mathrm{particle}\;\mathrm{surface}\;\mathrm{area}\right)∼1/v\cdot v^{2/3}∼v^{-1/3}∼\kappa$. From Equation (8), it is clear that ϕ and κ have exactly the same effect (NSA is usually correlated with the primary particle size. As argued before, we think of primary or secondary aggregates to screen cracks. However, there is a strong correlation between primary aggregate and primary particle size; see, e.g., [39]). Nevertheless, we plot a variation of κ around the fit value for different temperatures and ϕ=0.2 in Figure 5b. We would like to add that it is not clear whether *v* corresponds to the volume of primary or secondary carbon black aggregates, i.e., a non-breakable assembly of primary particles or a weakly bonded collection of primary aggregates. The latter would be certainly broken at the point of rupture. However, even a non-bonded, but densely packed collection of carbon black would possibly be able to screen a crack.

Our model also allows investigating the influence of deformation speed *v* on tensile strength. As already stated, for unfilled rubbers, temperature and speed are a function of $t/a_{T}$ only, i.e., they obey the time–temperature superposition principle. For filled rubbers, matters are more complex. Originally, we introduced deformation speed via the timescale *t* in Equation (3), which we set constant. A faster speed corresponds to a smaller timescale *t*. Equivalently, as our model is solely based on a competition between the timescale of deformation and the rate of defect/crack propagation, we could reduce the rate constants. We did so by replacing $r_{d,0}\to r_{d,0}v_{0}/v$ and $r_{b,0}\to r_{b,0}v_{0}/v$ in Equation (13), where $v_{0}$ is the speed used for fitting. The result for ϕ=0.2 is presented in Figure 6b. Reference [31] used 50 phr high-abrasion furnace black (comparable to N339) in SBR and observed an increase of about 30% in tensile strength over three decades of velocity at 25 °C. This is in the same order as our predictions.

TSSR measurements were successfully used to investigate the structure of sulfur bonds in various elastomers [32,40]. This kind of measurement was also performed on the samples published in [20], where sample “ref” was exactly of the same composition as our 50 phr sample. For the latter, TSSR measurements are presented in Figure 4a. The sample codes “1250 °C” and “2500 °C” correspond to (partially) graphitized samples with reduced surface activity; see [20]. All samples were held at 100% strain while the temperature was increased from around 23 °C to 200 °C by 2 °C/min. We present the raw data, viz. no correction for thermal expansion or entropy elasticity was performed. After the experiment and cooling down, the samples appeared to be “re-crosslinked” such that their equilibrium state was seemingly the stretched one. For this reason, we can assume that under these experimental conditions, the network did not break down catastrophically, but the sulfur bonds were certainly broken (and reformed, due to the “gentle” procedure). Thus, we can apply our theory to the TSSR data. Certainly, the TSSR measurement was carried out on a longer timescale than the stretching experiments (total heating time ≈100 min), but the exact conversion factor remains unknown. The dashed line in Figure 4a represents the model prediction using a rate conversion factor of six. The agreement with the experiment is excellent once we reach the regime of supposedly temperature-limited stress. We would like to highlight that even the slope was captured well, and it is not at all self-evident that this could be achieved by only adjusting the timescale. The slope is sensitive to the energy barrier $E_{b}$, and thus, the choice of 80kJ/mol is somehow justified by the result. Finally, in Figure 4b, we present the same model prediction, but by including only the defect rate $r_{d}$ and bond-breaking rate $r_{b}$ in Equation (13). Obviously, bond breaking is the dominating effect in the temperature range above 130 °C.

## 5. Conclusions

We presented experimental data regarding the temperature and filler-dependent tensile strength of EPDM rubber, which was explained by screening of crack nuclei at filler particles and force-induced breakdown of cross-links. The model’s predictions are in full accordance with the literature with respect to the variation of the deformation speed and data on temperature scanning stress relaxation measurements. However, the influence of varying cross-link density remains obscure.

If the model is correct, tensile strength for “simple” (e.g., non-crystallizing) rubber is a function of glass transition temperature, filler amount, and filler surface area and relatively (see the discussion above) independent of polymer–filler coupling. The model should be validated using other polymers, especially with differing glass transition temperatures or polymers with softeners. This could also help elucidate the validity of Equation (7). Finally, the hypothesis of breaking sulfur bonds could be challenged by applying the model to a differently cross-linked material, e.g., a peroxide- or radiation-cured one.

## Figures and Tables

**Table 1:** Parameters of the model. The procedure used to obtain them is described in the text.

Name	Value	Unit	Origin
$c_{1}$	1.13	1	Literature [20]
$c_{2}$	85.3	K	Literature [20]
$T_{r}$	276.15	K	Literature [20]
α	0.42±0.05	1	Fit ϕ=0 (Equation (8))
$r_{d,0}$	0.28±0.15	1	Fit ϕ=0 (Equation (8))
$E_{b}$	80	kJ/mol	Literature [20,37]
*V*	$\left(4.6\pm 0.6\right)\times 10^{-4}$	m³/mol	Fit σ-plat. (Equation (11))
$r_{b,0}$	$\left(8\pm 2\right)\times 10^{7}$	1	Fit σ-plat. (Equation (11))
κ	48±2	1	Fit Equation (13)

## Data Availability

Not applicable.
